# Influence of the oxide layer for growth of self-assisted InAs nanowires on Si(111)

**DOI:** 10.1186/1556-276X-6-516

**Published:** 2011-08-31

**Authors:** Morten Hannibal Madsen, Martin Aagesen, Peter Krogstrup, Claus Sørensen, Jesper Nygård

**Affiliations:** 1 Nano-Science Center, Niels Bohr Institute, University of Copenhagen, 2100 Copenhagen, Denmark; 2 SunFlake A/S, Nano-Science Center, Universitetsparken 5, 2100 Copenhagen, Denmark

## Abstract

The growth of self-assisted InAs nanowires (NWs) by molecular beam epitaxy (MBE) on Si(111) is studied for different growth parameters and substrate preparations. The thickness of the oxide layer present on the Si(111) surface is observed to play a dominant role. Systematic use of different pre-treatment methods provides information on the influence of the oxide on the NW morphology and growth rates, which can be used for optimizing the growth conditions. We show that it is possible to obtain 100% growth of vertical NWs and no parasitic bulk structures between the NWs by optimizing the oxide thickness. For a growth temperature of 460°C and a V/III ratio of 320 an optimum oxide thickness of 9 ± 3 Å is found.

## 1 Introduction

Nanowires (NWs) can potentially improve the efficiency of devices, e.g., in photonics [1], energy storage [2], bio sensing [3], and high-speed electronics [4]; and most likely such applications will require integration with silicon-based platforms. For some of the applications, a high density of uniform NWs without any parasitic growth is needed. The vast majority of NW growth research has been using Au as the collector particle. Recently, self-assisted NW growth of both GaAs and InAs on Si(111) has been reported for MBE directly on oxide [5-8], from e-beam lithography defined holes in the oxide layer [9-11] and on bare substrates [12], and also, self-assisted InAs NW growth by MOCVD has been reported [13-15].

For this study, we concentrate on growth of self-assisted InAs NWs, since InAs NWs have superior properties for electron transport devices compared to most other III-V materials [4]. It is furthermore of great interest to combine the properties of III-V materials with the well-established silicon technology; but this requires a completely gold-free environment, as gold is known to be detrimental to the opto-electronic properties of silicon.

## 2 Growth of self-assisted NWs

All NWs in this study were grown on 2-inch epiready undoped Si(111) substrates using a solid source Varian GEN II molecular beam epitaxy (MBE) system. The substrates were pre-degassed at 500°C before transfer into the growth chamber where they were degassed for 8 min at 630°C immediately before growth. The temperature was then lowered to 460°C, and the growth was initiated by opening the In-shutter. The beam equivalent pressure (BEP) was measured using an ion gauge and growth rate calibrations were performed using reflection high-energy electron diffraction (RHEED). We used an In BEP of 4 × 10^-8 ^torr, corresponding to a bulk InAs growth rate of 100 nm/h. The As flux was turned on during the cool down from annealing to growth temperature unless otherwise stated. No pure In deposition was necessary for initializing growth, similar to the case of GaAs NW growth on Si(111) [6].

The exact growth mechanism is still unclear, and both vapor-liquid-solid [16] and vapor-solid [8] have been reported for self-assisted InAs. The growth is initiated either by the formation of openings in the oxide [16] or by dissolution of oxide by group III materials at the droplet/substrate interface, giving rise to a vapor-liquid-solid growth mechanism. GaAs NW growth has been demonstrated on SiO_2 _layers with a thickness of up to 30 nm [5], whereas a much thinner layer is required for InAs.

**Figure 1:**
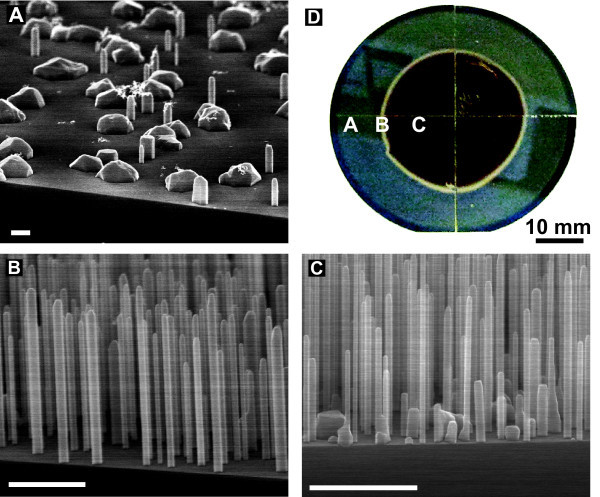
**Nanowires grown on Ga-deoxidized substrate**. **(A-C) **Sideview scanning electron microscope (SEM) images of the three different wafer positions as marked in **(D)**, corresponding to different oxide layer thicknesses. **(D) **An optical image of a full 2-inch wafer where the oxide has only been removed in the outer part. Area **(A) **is an example of growth regime 3 and areas **(B, C) **are from growth regime 2 (see text). The absence of parasitic bulk structures makes area **(B) **superior to area **(C)**. White scale bars are 1 *μ*m.

The key parameters to control the NW morphology, length, and width have been reported to be the temperature and the incoming fluxes, especially the V/III-ratio [7,10,17]. For self-assisted InAs NWs, the pre-treatment of the substrate was also observed to play a crucial role for obtaining high-quality growth results. On the basis of our results using different pre-treatment techniques, we have found that the oxide layer thickness is a critical parameter for controlling the density and yield. In general, the NW growth can be divided into three different types of morphologies: (1) Growth on oxide; high density, and many tilted NWs; (2) Growth on a thin oxide layer (approx. 1 nm); vertical and high aspect ratio NW growth (see Figure 1B,C); and (3) Growth without oxide; vertical NW growth, with a low density and low aspect ratio and high probability of parasitic structures (Figure 1A). We have in particular focused on the second regime, as it seems to be the most promising for growth of NWs.

The high lattice mismatch (≈12%) between InAs and Si does not suppress the growth of NWs, and in regime 2 and 3 NWs only grow perpendicular to the substrate. We have investigated the influence of an As flux at different stages in the growth process, i.e., before and during annealing, in the cool down time to growth temperature, simultaneously with the In flux and a few seconds after the In flux. No differences in the amount of vertical NWs were found. This observation is much different than for growths using MOCVD, where advanced cool down procedures has been developed for obtaining vertical NWs [15]. A theoretical study by Koga describes how pre-adsorption of first As and then In assists the formation of a coherent surface and makes it possible to grow vertical NWs [18]. The difference between the two growth systems might be due to the necessary pre-cracking in an MOCVD growth system, or because of residuals from the cracking that affect the substrate surface. Furthermore, the growth temperature is lower in MBE which gives a lower solubility of Si in In [19].

## 3 Study of the oxide layer

All substrates are covered by a native oxide layer. Using spectroscopic ellipsometry we have measured the oxide layer thicknesses to (14 ± 1) Å for substrates taken ^_ ^directly from the box.

The oxide layer can be removed by hydrofluoric acid (HF) which simultaneously passivates the surface, preventing formation of a new oxide, at least for the short time it takes to load the sample and evacuate the chamber [20]. Only areas in direct contact with the HF will get deoxidized, making it possible to remove the oxide from only a part of the substrate.

To ensure the removal of the oxide without contaminating the substrate, we employed Ga-assisted deoxidization [21]. SiO_2 _desorbs at temperatures around 900°C depending on the composition and background pressure. Ga can react with silicon oxide via the chemical reactions [22]

(1) $$\text{Si}{\text{O}}_{2}+4\text{Ga}\to \text{Si}+2\text{G}{\text{a}}_{2}\text{O}$$

(2) $$\text{Si}{\text{O}}_{2}+2\text{Ga}\to \text{SiO}+\text{G}{\text{a}}_{2}\text{O}$$

and the excess Si reacts further via

(3) $$\text{Si}{\text{O}}_{2}+\text{Si}\to 2\text{SiO}$$

Both SiO and Ga_2_O desorb at a much lower temperature than SiO_2_. From the stoichiometry, we can expect to remove one SiO_2 _pair for every two Ga atoms, and as the lattice spacing for SiO_2 _and GaAs is almost identical, we can use the bulk growth rate for GaAs measured with RHEED to get an estimation of the evaporation rate.

Using ellipsometry, we have measured the deoxidization rate. After unloading the sample from the MBE system, and exposing it to air, it was transferred immediately to the ellipsometer. We have corrected the data for the small amount of reoxidization in the transfer period. We find that the deoxidization rate is slightly larger than expected from the stoichiometric calculations, i.e., deposition of the amount of Ga to form a 2-nm GaAs bulk layer removes slightly more than 1-nm SiO_2_. This is close to the result by Wright and Kroemer who state that the deoxidization rate is slightly smaller than the stoichiometric amount [21]. The difference might be due to the native oxide layer consisting of SiO_*x*_, where *x *is a number between 1 and 2, which will increase the desorption rate.

The Ga-deoxidization reactions are very temperature sensitive around 800°C [21]. By exploiting the substrate temperature gradient when growth is carried out without a backside diffuser plate, we were able to make a partial deoxidization. The oxide layer has only been removed in the hotter part of the substrate, recognized as the bright part of the optical image in Figure 1D. We used a Ga deposition rate equivalent to a bulk growth rate of GaAs of 300 nm/h and a temperature of 820°C, measured with a pyrometer. The Ga flux was on for 30 min and afterward the substrate was kept at 820°C for 10 min to ensure that all Ga was re-evaporated. As control experiments, we have raised the temperature to 840°C, which completely deoxidizes the entire substrate, and second heat up the substrate without applying Ga, giving no measurable deoxidization.

## 4 Substrate temperature gradient

A pyrometer averages the measured temperature over a larger area; and to get a more thorough understanding of the substrate temperature gradient, we have made simulations using the software COMSOL Multiphysics. The modeling is based on the geometry of the MBE substrate mount. The MBE system is designed to handle 3-inch substrates, but for this study we use an insert to the holder for 2-inch substrates. The substrate is heated by thermal radiation from the backside of the holder. A thermocoupler is placed in the center of the heater, but this did not affect the simulations, so instead a homogenous radiation is assumed over both the substrate and holder.

The emissivity, *ε*, is a measure of a given materials' ability to emit energy by radiation. For undoped silicon, the emissivity is highly temperature dependent, a value of *ε*_Si _= 0.2 is used for the growth temperature and *ε*_Si _= 0.7 is used for the temperature for Ga-deoxidization [23]. Both the holder and the insert to the holder are made of molybdenum. For this material, the total emissivity is more constant in the growth temperature regime, and values of *ε*_Mo _= 0.09 and *ε*_Mo _= 0.12 are used for the growth and Ga-deoxidization temperature, respectively [24].

**Figure 2:**
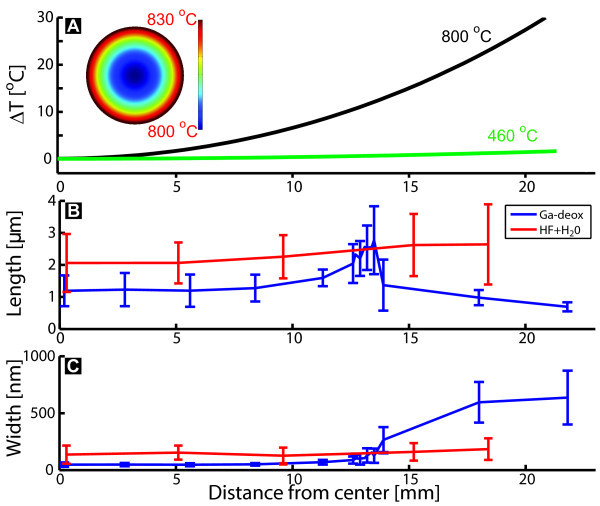
**Temperature and NW morphology across a 2-inch substrate**. **(A) **Simulation of the temperature during Ga-deoxidization and growth. Inset shows the full wafer. **(B, C) **Morphology of NWs for two pre-treatment methods as a function of the radial distance from the center. The blue curve is data from the growth with Ga-deoxidization shown in Figure 1 and the red curve is an HF deoxidized substrate with similar growth conditions. The longest NWs grown on the Ga-deoxidized substrate is observed to be at position B marked in Figure 1. The growth time is 60 min and an As_4 _BEP of 1.30 × 10^-5 ^torr, corresponding to a V/III-ratio of 320 has been used for both substrates.

The simulation is solved numerically in three dimensions using finite-element analysis for a steady-state system. The simulated temperature gradients on the surface of the substrates are shown in Figure 2A and the inset shows a surface plot of a substrate at the Ga-deoxidization temperature. At a substrate temperature of 460°C the temperature gradient is seen to be less than 2°C, having little effect on the growth conditions, whereas the gradient is 30°C at the Ga-deoxidization temperature, affecting the local deoxidization efficiency.

## 5 Comparison of deoxidization methods

For similar growth conditions, two deoxidization methods are compared in Figure 2B,C. The blue curve is for the same growth as shown in Figure 1 where the Ga-deoxidization method is used, whereas the red curve is for a substrate dipped in 5% HF for 10 s and rinsed with Millipore water (*>*18 M*Ω *resistance) for 1 min, which forms a thin oxide layer. The average width and height of the NWs are plotted in Figure 2B,C as a function of the radial distance from the center of the wafer. It shall be emphasized that the temperature gradient in Figure 2A only applies for the Ga-deoxidized substrate during the deoxidization process. All NWs for both deoxidization methods are observed to grow perpendicular to the substrate and therefore belonging to either regime 2 for a thin oxide layer or regime 3 in areas where the oxide has been completely removed.

The width and length distributions are highly uniform across the HF-etched substrate, whereas for Ga-deoxidized it completely changes around 13 mm from the center. This area is recognized as the bright band in Figure 1D. In this band, the length and width distributions of the NWs are similar to the ones from the HF-etched substrate and no parasitic bulk structures in between the NWs are found (see Figure 1B). This growth regime is therefore of paramount interest for self-assisted InAs NWs. To our knowledge, the results above are the first report of parasitic island free growth of self-assisted NWs on non-pre-patterned substrates.

The large variation of the lengths and widths within the same area, represented by the error bars, may be explained by the formation of non-uniform openings in the oxide film. Mandl et al. [16] has measured openings in a SiO*_x _*layer on InAs(111)B ranging from less than 100 nm to several micrometers. In the oxide-free areas, the morphology of the NWs is very different, and a low density of thick and short NWs are found. This clearly shows that the oxide layer plays a major role for self-assisted NW growth.

**Figure 3:**
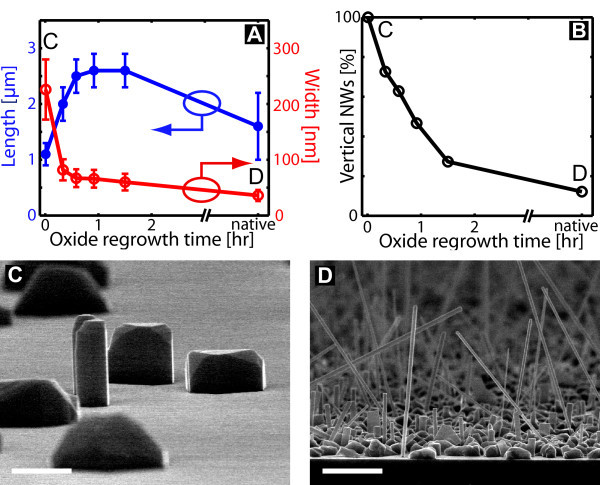
**Regrowth of oxide on a 200°C hotplate**. **(A) **Length and width of NWs as a function of regrowth time for the oxide layer. **(B) **Percentage of vertical NWs, indicating an epitaxial relation to the substrate. The As_4 _flux is 1.30 × 10^-5 ^torr, corresponding to a V/III-ratio of 320, and the growth time is 30 min. The point to the left marked with a C is without any re-oxidization treatment. A typical SEM image for this regime is shown in **(C)**. **(D) **SEM image of growth on a native oxide layer marked with a D in the graphs. Scale bars are 1 *μ*m.

Another pre-processing approach is to remove the oxide layer completely by HF and then regrow the oxide layer. The latter was done by placing the substrate on a 200°C hotplate in a fumehood, similar to the experiment performed in [6]. For non-treated substrates. we observe the growth of NWs in many different directions (Figure 3D), defined as growth regime 1 above, indicating a non-epitaxial growth with respect to the substrate. For growth on completely oxide-free wafers, similar to Figure 1A, only vertical NWs are observed showing that no other (111) facets have been formed between NWs and substrate during growth initialization (Figure 3C).

The data shown in Figure 3 are obtained from growth on the same substrate, by careful etching part of it at different times in the pre-processing. The aforementioned temperature gradient is much smaller at the growth temperature, so the data can be compared directly. Even a few minutes on the hotplate seems sufficient to destroy the hydrogen passivation and thereby creating an oxide layer.

The average length and width of the NWs as a function of oxide regrowth time reach a fairly constant level almost immediately (Figure 3A), whereas the yield of vertical NWs drop with re-oxidization time (Figure 3B). Another growth with re-oxidation times ranging from 2 to 26 h indicates that the yield of vertical NWs is constant after around 90 min. The re-growth of oxide on a hotplate seems less favorable than the other methods investigated above because of the high fraction of non-vertical NWs.

## 6 Conclusion

In conclusion, we have shown that focus should also be put on the oxide layer thickness and that the substrate preparation is important for self-assisted growth of InAs NWs. It is found that the growth regime giving the longest NWs with the fewest parasitic bulk structures is achieved for an oxide layer thickness between the native oxide and no oxide. More precisely we have found that an oxide layer of 9 ± 3 Å gives the best results for our growth parameters. Moreover, several methods are used to control the oxide layer thickness and we have shown that the ultra clean method of Ga de-oxidation gives the best results. We believe this is because the completely impurity free environment and this therefore demonstrates a new route toward obtaining perfect NW growth on an entire substrate surface.

## Abbreviations

BEP: beam equivalent pressure; HF: Hydrofluoric acid; MOCVD: metal organic chemical vapor deposition; MBE: molecular beam epitaxy; NW: nanowire; RHEED: reflection high-energy electron diffraction; SEM: scanning electron microscopy.

## Competing interests

The authors declare that they have no competing interests.

## Authors' contributions

MHM designed and carried out the experiments and drafted the manuscript. MA assisted the design of the experiment, participated in the discussion of the results and in revising the manuscript. PK participated in the discussion of the results and in revising the manuscript. CBS and JN supervised the study and revised the manuscript. All authors read and approved the final version of the manuscript.
